# Assessment of Primary Care Appointment Times and Appropriate Prescribing of Statins for At-Risk Patients

**DOI:** 10.1001/jamanetworkopen.2021.9050

**Published:** 2021-05-11

**Authors:** Allison J. Hare, Srinath Adusumalli, Saehwan Park, Mitesh S. Patel

**Affiliations:** 1 Penn Medicine Nudge Unit, University of Pennsylvania, Philadelphia; 2 Perelman School of Medicine, University of Pennsylvania, Philadelphia; 3 Crescenz Veterans Affairs Medical Center, Philadelphia, Pennsylvania; 4 Wharton School, University of Pennsylvania, Philadelphia

## Abstract

This cohort study examines whether there is an association between primary care appointment times and statin prescribing rates for patients with elevated risk of major adverse cardiovascular events.

## Introduction

Cardiovascular disease is the leading cause of adult morbidity and mortality in the United States and globally.^1^ Guideline-directed statin therapy has been demonstrated to reduce the risk of major adverse cardiovascular events,^2^ yet half of statin-eligible patients have not been prescribed one.^3^ In prior work, we observed that rates of preventive care including influenza vaccination and cancer screening declined as the clinic day progressed.^4,5^ It is unknown whether this pattern exists with statin prescribing. Our objective was to evaluate the association between primary care appointment times and statin prescribing rates for patients with elevated risk of major adverse cardiovascular events.

## Methods

This cohort study was approved by the University of Pennsylvania institutional review board with a waiver for consent because the study posed minimal risk and because it was infeasible given the retrospective study design. We followed the Strengthening the Reporting of Observational Studies in Epidemiology (STROBE) reporting guideline.

Clarity, an Epic reporting database, was used to obtain data from 28 Penn Medicine primary care practices. We included each patient’s first new or return visit with their primary care practitioner (PCP) March 1, 2019, to February 29, 2020. Statin eligibility was based on the United States Preventive Services Task Force guidelines, presence of clinical atherosclerotic cardiovascular disease (ASCVD) or familial hypercholesterolemia diagnosis, or low-density lipoprotein cholesterol greater than or equal to 190 mg/dL (to convert to millimoles per liter, multiply by 0.0259). Patients were excluded if they were already taking a statin or had documented statin intolerance. Race/ethnicity was self-reported. The primary outcome measure was presence of a statin prescription on the day of the visit.

Appointments from 8:00 am to 4:59 pm were grouped by the hour. In the adjusted analysis, we fit a conditional logistic regression conditioned on PCP consistent with prior work.^5^ We used this model because it stratified the analysis by clinician, thereby avoiding comparing clinicians who only practice in the afternoon with those who only practice in the morning. Models were adjusted for patient demographic characteristics (age, sex, race/ethnicity, and income), insurance type, Charlson Comorbidity Index, clinical ASCVD diagnosis, appointment type, and fixed effects by practice and month. In addition to covariates for each hour, models were fit to compare morning (8:00 am to 11:59 am) vs afternoon (12:00 pm to 4:59 pm) appointments and a continuous time variable. Two-sided hypothesis tests used a significance level of .05, and analyses were conducted using Python version 3.8.6 (Python Software Foundation) and the statsmodels package version 0.12.1. Statistical analysis was performed from November 2020 to February 2021.

## Results

The sample comprised 10 757 patients with a mean (SD) age of 66.0 (10.5) years; 5072 (47.2%) were female, and 7071 (65.7%) were White. Patient characteristics were similar between morning and afternoon appointments. Overall, statins were prescribed in 3864 visits (35.9%). Statins were prescribed in 2447 morning visits (37.5%) and 1417 afternoon visits (33.4%) (Figure). Compared with 8 am (reference group), the adjusted odds ratio (aOR) of statin prescribing was significantly lower at all hours except 9 am (eg, 9 am: 0.88 [95% CI, 0.76-1.02]; *P* = .10; 10 am: 0.85 [95% CI, 0.73-0.99]; *P* = .04; 12 pm: 0.63 [95% CI, 0.49-0.83]; *P* = .001; 3 pm: 0.69 [95% CI, 0.57-0.82]; *P* < .001). Compared with morning appointments, the odds of statin prescribing during afternoon appointments were significantly lower (aOR, 0.79; 95% CI, 0.73-0.87; *P* < .001). The overall time trend for statin prescribing decreased significantly (aOR, 0.95; 95% CI, 0.94-0.97; *P* < .001) (Table).

**Figure.**
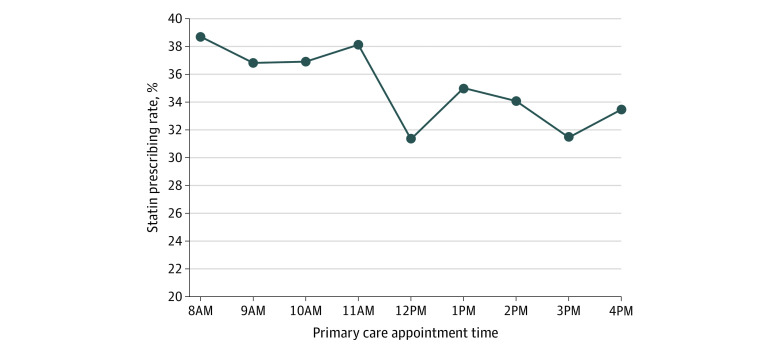
Statin Prescribing Rates by Primary Care Appointment Time Unadjusted data are from March 1, 2019, to February 29, 2020, and are based on each patient’s first new or return visit with their primary care practitioner. Primary care appointment times are grouped by the start of each hour (eg, 8:15 am and 8:30 am were placed in the 8 am group).

**Table.** Adjusted Odds of Statin Prescribing by Primary Care Appointment Time

Primary care appointment time	Adjusted OR (95% CI)^a^	*P* value
Morning		
8 am	1 [Reference]	NA
9 am	0.88 (0.76-1.02)	.10
10 am	0.85 (0.73-0.99)	.04
11 am	0.80 (0.67-0.94)	.008
Afternoon		
12 pm	0.63 (0.49-0.83)	.001
1 pm	0.73 (0.62-0.87)	<.001
2 pm	0.70 (0.59-0.82)	<.001
3 pm	0.69 (0.57-0.82)	<.001
4 pm	0.71 (0.56-0.89)	.003
Afternoon vs morning^b^	0.79 (0.73-0.87)	<.001
Overall time trend^c^	0.95 (0.94-0.97)	<.001

Abbreviations: NA, not applicable; OR, odds ratio.

^a^ Adjusted ORs represent the relative odds of statin prescribing for each hour after 8 am (reference group). Appointment times were grouped by the start of the hour (eg, 8:15 am and 8:30 am were placed in the 8 am group).

^b^ Afternoon vs morning uses an adjusted model with a dichotomous variable for appointment time with 0 for appointments 8:00 am to 11:59 am (reference group) and 1 for appointments 12:00 pm to 4:59 pm.

^c^ Overall time trend uses an adjusted model with a continuous variable for appointment time with 1 equal to 8 am and 9 equal to 4 pm. The adjusted ORs represent the relative odds of statin prescribing for each incremental 1-hour period. For example, an adjusted OR of 0.95 can be interpreted as 5% lower odds per hour for each hour after 8 am.

## Discussion

Among a primary care network of 28 practices, statin prescribing decreased significantly as the day progressed, particularly in the afternoon relative to the morning. To our knowledge, this is one of the first studies to evaluate statin prescribing variations by appointment time. These findings are likely multifactorial in nature. First, as the day progresses clinicians may run behind schedule which can lead to rushed interactions, decreased bandwidth for discussions of indicated therapies, or implicit agreements to defer complex decisions.^6^ Second, as the day progresses, clinicians and patients are more likely to face decision fatigue, or the erosion of initiative that results from the cumulative cognitive burden of a long session of decision-making.^4,5^

This study was limited by its observational design at one health system. Future interventions targeting improvements in statin prescribing should consider the influence of time of day.
